# Factors Affecting Korean Medicine Health Care Use for Functional Dyspepsia: Analysis of the Korea Health Panel Survey 2017

**DOI:** 10.3390/healthcare10071192

**Published:** 2022-06-25

**Authors:** Boram Lee, Changsop Yang, Mi Hong Yim

**Affiliations:** 1 KM Science Research Division, Korea Institute of Oriental Medicine, Yuseong-daero 1672, Yuseong-gu, Daejeon 34054, Korea; qhfka9357@kiom.re.kr (B.L.); yangunja@kiom.re.kr (C.Y.); 2 Digital Health Research Division, Korea Institute of Oriental Medicine, Yuseong-daero 1672, Yuseong-gu, Daejeon 34054, Korea

**Keywords:** functional dyspepsia, Korean medicine, health care use, Korea Health Panel Survey, Andersen’s behavioral model

## Abstract

Functional dyspepsia (FD) significantly reduces quality of life, and Korean medicine treatment, including herbal medicine, is frequently used in the clinical setting. We aimed to analyze the factors affecting Korean medicine health care (KMHC) use for FD. Data from the Korea Health Panel Survey 2017 were analyzed. Individuals aged > 19 years who were diagnosed with FD and used outpatient care were included. Multiple logistic regression analyses were performed to investigate the association of predisposing, enabling, and need factors with KMHC use for FD, based on Andersen’s behavioral model. The best subsets of factors affecting KMHC use for FD were selected using a stepwise procedure. Participants aged 65 years or older were less likely to use KMHC to treat FD than those aged 19 to 34 years (odds ratio (OR), 0.14; 95% confidence interval (CI), 0.02–0.93). Residents of Busan, Daegu, Ulsan, or Gyeongsang tended to use more KMHC to treat FD than those of Seoul, Gyeonggi, or Incheon (OR, 2.45; 95% CI, 1.02–5.88). Participants with private health insurance were more likely to use KMHC to treat FD than those without private health insurance (OR, 3.41; 95% CI, 1.02–11.42). The prediction model of KMHC use for FD selected sex, age, private health insurance, and stress as the best subset of factors (AUC, 0.709; 95% CI, 0.637–0.781). The results of this study will aid in the decision making of clinicians, researchers, and policymakers.

## 1. Introduction

Functional dyspepsia (FD) is a common disorder of the upper gastrointestinal tract. It is characterized by symptoms including postprandial fullness, early satiety, and epigastric pain or burning and has no clear structural cause [1]. The prevalence of FD is estimated to range from 10–30% worldwide [2]; in Korea, it is approximately 10.3% [3]. Although it is not a fatal disease, it incurs significant direct and indirect costs and lowers work productivity, resulting in a very large socioeconomic burden [4].

Conventional drugs, such as proton pump inhibitors and dopaminergic antagonists, have been used to relieve the symptoms of indigestion; however, there are limitations in treating FD because the disease has a complex pathophysiology and various symptoms. Therefore, the demand for complementary and integrative medicine is increasing globally [5]. In particular, herbal medicine, a representative modality of complementary and integrative medicines, has multi-component and multi-target properties; thus, targeting the various pathological mechanisms and symptoms of FD is possible [6]. A recent meta-analysis found that herbal medicine was more effective than prokinetic agents for alleviating global dyspeptic symptoms and concluded that it can be considered an alternative treatment for FD symptoms when prokinetic agents and proton pump inhibitors are contraindicated [7]. Therefore, especially in Korea, many people receive Korean medicine (KM) treatments, including herbal medicine and acupuncture, for FD, and data from the Korea Health Insurance Review and Assessment Service show that FD ranked as the eighth most common diagnosis among outpatients at KM institutions in 2021 [8].

An individual’s health level is closely related to their behaviors, which have an impact on medical use behavior. Therefore, the identification and analysis of medical use behavior can help promote individual health and the development of systems that can improve health and medical financial efficiency. Providing health care resources requires significant financial effort; therefore, knowledge of the determinants of health care use is important for health policy [9]. To date, studies related to KM treatment for FD have been conducted to identify the effects through clinical trials [10] and to check the status of herbal medicine prescriptions using data from the Health Insurance Review and Assessment Service [11]. To the best of our knowledge, studies examining the factors affecting KM health care (KMHC) use for patients with FD have not been conducted.

Andersen has developed a behavioral model describing the factors leading to health care use. This model has three categories of determinants of health service use: predisposing, need, and enabling factors [12]. It has been most widely used in health care utilization studies; several studies have used it to examine predictors associated with various health outcomes [9,13]. In this study, we sought to examine the status of KMHC use in patients with FD. Additionally, we examined the factors affecting KMHC use based on Andersen’s behavioral model.

## 2. Materials and Methods

### 2.1. Data source and Study Participants

This study was based on data obtained from the Korean Health Panel Survey (KHPS) 2017. The KHPS is a nationally representative panel survey that has been conducted annually by the Korea Institute for Health and Social Affairs (KIHASA) and the National Health Insurance Service (NHIS) since 2008. It comprises information on health care use, health care expenditure, and the various factors associated with them, which are socioeconomic status, demographic characteristics, private health insurance, health behaviors, and chronic diseases [14]. Data from the KHPS are obtained via questionnaire using a face-to-face computer-assisted personal interviewing (CAPI) method. The official website of KHPS provides microdata and further details (https://www.khp.re.kr, accessed on 1 October 2021).

**Figure 1:**
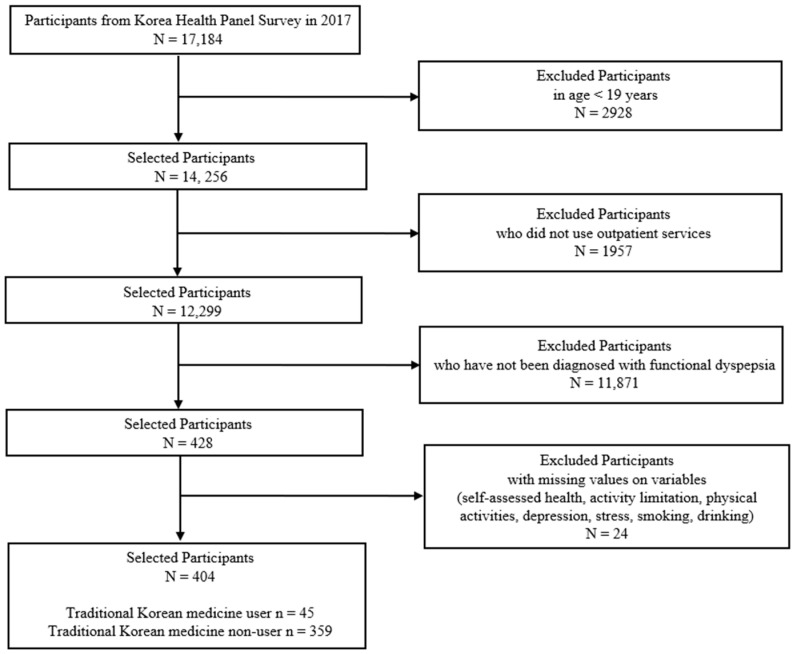
Flowchart of the sample selection procedure.

The participants of KHPS in 2017 consisted of 17,184 household members from 6408 households. Among those aged 19 years or older, we analyzed patients who had been diagnosed with FD (K30 code as the main diagnosis according to the International Classification of Diseases) and used outpatient care. Out of 17,184 participants, we sequentially excluded 2928 who were younger than 19 years old, 1957 who did not use outpatient care, 11,871 who had not been diagnosed with FD, and 24 with missing values for factors such as socioeconomic status, demographic characteristics, and health behaviors. Finally, 404 participants were included in our study (Figure 1). Approval for conducting the KHPS 2017 was obtained from the Institutional Review Board of the KIHASA. This study was approved for an exemption determination by the Institutional Review Board of the Korea Institute of Oriental Medicine (IRB No., I-2109/008-003).

### 2.2. Definitions

Participants diagnosed with FD were determined based on the questionnaire. The participants who answered the questionnaire item “If you used outpatient care, what was the main diagnosis you were diagnosed with?” with FD (K30 code according to the International Classification of Diseases and had one or more symptoms of postprandial fullness, early satiety, epigastric pain, or epigastric burning without organ findings that occurred at least six months prior and persisted for at least the previous three months [1]) were defined as participants diagnosed with FD.

KMHC use of FD was defined based on whether participants diagnosed with FD used KM outpatient care at least once. Therefore, among the participants who were diagnosed with FD and used outpatient care, those who used only KMHC or both KMHC and conventional Western medicine health care were classified as KMHC use for FD. Those who used only conventional Western medicine health care were classified as KMHC non-use for FD.

### 2.3. Measures

According to Andersen’s behavioral model, an individual’s use of health services relates to a predisposition that already exists regardless of the individual’s will to use health services, their ability to secure health services, and their perceived or diagnosed illness status. These refer to predisposing, enabling, and need factors, respectively [15]. Therefore, the variables that were considered as potential factors affecting health care use, such as socioeconomic status, demographic characteristics, private health insurance, health behaviors, and chronic diseases [12,16], were grouped into these three categories in this study.

The predisposing factors included sex, age, education, and region. Sex was dichotomized into men or women. Age was divided into four groups: 19–34 years, 35–49 years, 50–64 years, and 65 or older. Education level was trichotomized: elementary school or below, middle or high school, and college or above. Region was divided into five groups: Seoul/Gyeonggi/Incheon, Gangwon, Daejeon/Chungcheong/Sejong, Gwangju/Jeolla/Jeju, and Busan/Daegu/Ulsan/Gyeongsang.

The enabling factors consisted of household income, employment status, health insurance type, private health insurance, and number of household members. Household income was divided into quintiles in the range of 1st quintile (lowest) to 5th quintile (highest). Employment status was trichotomized into unemployed, employed, or self-employed. Health insurance type was divided into three groups: employee health insurance, local subscriber, and medical aid or others. Private health insurance was dichotomized according to whether the individual had taken out health insurance privately or not. The number of household members was divided into four groups: 1, 2, 3, and 4 or more.

The need factors comprised disability, self-assessed health, number of chronic diseases, depressed mood, stress, body mass index (BMI), smoking status, drinking, and physical activities. Disability was dichotomized depending on its presence or absence. Self-assessed health was trichotomized into poor, fair, or good. The number of chronic diseases was divided into four groups according to the number of diagnosed diseases among hypertension, diabetes, hyperlipidemia, arthropathy, heart disease (including heart failure, chronic rheumatic heart diseases, pulmonary heart disease, pericarditis, endocarditis, and myocarditis), ischemic heart disease, cerebrovascular disease, or malignant neoplasm: 0, 1, 2, and 3 or more. Depressed mood was dichotomized into yes or no according to whether the individual had felt depressed for at least two consecutive weeks in the past year. Stress was divided into three groups according to frequency: never or rarely, sometimes, and frequently or always. BMI was divided into five groups according to Asian-Pacific guidelines: <18.5, 18.5–22.9, 23.0–24.9, 25.0–29.9, and ≥30 [17]. Smoking status was trichotomized into never smoked, quit smoking, or smoking. Drinking was divided into four groups according to frequency of alcohol consumption: never drunk, monthly or less, 2 to 4 times a month, and 2 times a week or more. Physical activities were dichotomized: not at all and once a week or more.

### 2.4. Statistical Analysis

All statistical analyses were performed using R version 4.0.3 (R Foundation for Statistical Computing, Vienna, Austria). The significance level of all statistical tests was set at 0.05. To examine differences in the individual predictors of predisposing, enabling, and need factors between the KMHC use and non-use groups, Fisher’s exact tests and chi-squared tests were conducted. Fisher’s exact tests were used when one of the cells in the contingency tables had less than five observations; otherwise, chi-squared tests were used. The results are presented as frequencies and percentages.

Simple logistic regression analyses were conducted to evaluate the association of the individual predictors of predisposing, enabling, and need factors with KMHC use for FD. Next, multiple logistic regression analyses were performed to assess the relative effect of predisposing, enabling, and need factors on KMHC use for FD by adding the three categories’ factors sequentially. Odds ratios (ORs) and 95% confidence intervals (CIs) were presented as the results of the logistic regression analyses. The mean of generalized variance inflation factors (mean GVIF) was used to confirm multicollinearity among a subset of predictors in multiple logistic regression analyses [18].

To identify the best subsets of factors affecting KMHC use in patients with FD and the best fit model, stepwise procedures based on the Akaike information criterion (AIC) were applied to the models by sequentially adding predisposing, enabling, and need factors. Three models were constructed. Model 1 consisted of factors selected by a stepwise procedure after entering all of the predisposing factors. Model 2 consisted of factors selected by a stepwise procedure after entering all of the predisposing and enabling factors. Model 3 consisted of factors selected by a stepwise procedure after entering all of the predisposing, enabling, and need factors. The AIC and the receiver operating characteristic (ROC) curve analysis were adopted to evaluate model performance. The results are presented as selected predictors and 5-fold cross-validated [19,20,21] areas under the ROC curve (AUC values) with 95% CIs.

## 3. Results

### 3.1. General Participant Characteristics

Of the 404 participants, 45 used KM outpatient care at least once after they were diagnosed with FD in 2017. Significant differences were observed in age, education, household income, private health insurance, and number of chronic diseases between KMHC use and non-use groups. The proportion (35.56%) of participants aged 50–64 years was highest in the KMHC use group. By contrast, the proportion (52.92%) of those aged 65 years or older was highest in the KMHC non-use group. Regarding education, the proportion (15.56%) of less-educated participants who graduated only elementary school or below was the lowest in the KMHC use group; the proportion (16.99%) of highly educated participants who graduated college or above was lowest in the KMHC non-use group. The proportions (4th quintile, 31.11%; 5th quintile, 26.67%) of participants with high household income tended to be high in the KMHC use group; the proportions (1st quintile, 26.18%; 2nd quintile, 25.91%) of those with low household income tended to be high in the KMHC non-use group. The proportion of participants with private health insurance and those without chronic diseases was higher in the KMHC use group (private health insurance, 86.67%; chronic diseases, 53.33%) than in the KMHC non-use group (private health insurance, 63.51%; chronic diseases, 32.31%). There were no significant differences in sex, region, employment status, health insurance type, number of household members, disability, self-assessed health, depressed mood, stress, BMI, smoking, drinking, and physical activities between the KMHC use and non-use groups (Table 1).

### 3.2. Factors Affecting KMHC Use for FD

The crude analysis, including only one individual predictor among predisposing, enabling, and need factors, showed that KMHC use was significantly related to age, education, household income, private health insurance, and number of chronic diseases, respectively. These significant associations changed a little in the analyses that were adjusted by adding predisposing, enabling, and need factors sequentially. In the adjusted analysis that included predisposing factors, age and region were significantly associated with KMHC use. Participants aged 65 years or older were less likely to use KMHC to treat FD when compared with those aged 19–43 years (OR, 0.19; 95% CI, 0.05–0.7). Residents of Busan, Daegu, Ulsan, or Gyeongsang tended to use more KMHC to treat FD than residents of Seoul, Gyeonggi, or Incheon (OR, 2.29; 95% CI, 1.06–4.92). In the adjusted analysis that included predisposing and enabling factors, there was a significant association between KMHC use and region. The likelihood of KMHC use to treat FD in residents of Busan, Daegu, Ulsan, or Gyeongsang was similar to the adjusted analysis that included predisposing factors alone (OR, 2.53; 95% CI, 1.14–5.61). After adjustment for the enabling factors to the model that included predisposing factors, the association between age and KMHC use became non-significant. In the fully adjusted analysis that included the predisposing, enabling, and need factors, age, region (of the predisposing factors), and private health insurance (of the enabling factors) were significantly associated with KMHC use. By contrast, no need factors were associated with KMHC use. Participants aged 65 years or older tended to use less KMHC to treat FD when compared with those aged 19–34 years (OR, 0.14; 95% CI, 0.02–0.93). Residents of Busan, Daegu, Ulsan, or Gyeongsang were more likely to use KMHC to treat FD when compared with residents of Seoul, Gyeonggi, or Incheon (OR, 2.45; 95% CI, 1.02–5.88). Participants with private health insurance were more likely to use KMHC to treat FD when compared with those without private health insurance (OR, 3.41; 95% CI, 1.02–11.42). The mean GVIF values of the adjusted analyses ranged from 1.161–1.547 where multicollinearity was not detected (Table 2).

### 3.3. Predictive Powers of the Predisposing, Enabling, and Need Factors for KMHC Use to Treat FD

The best subsets of factors affecting KMHC use were selected based on the AIC of stepwise procedures. The AUC values were calculated using 5-fold cross-validated areas.

In model 1 for predisposing factors, sex, age, and region were selected. In model 2 for predisposing and enabling factors, sex, age, region, and private health insurance were selected. In model 3 for predisposing, enabling, and need factors, four predictors were selected: sex, age, private health insurance, and stress. Model 3 had the lowest AIC value among the three predictive models; the lower the AIC value is, the higher the model quality (model 1, AIC, 273.127; model 2, AIC, 272.558; model 3, AIC, 264.762). The AUC values of the three models ranged from 0.696 to 0.709. The AUC value of model 3 was the highest (model 1, AUC, 0.701; 95% CI, 0.626–0.777; model 2, AUC, 0.696; 95% CI, 0.623–0.768; model 3, AUC, 0.709; 95% CI, 0.637–0.781). *p*-values were calculated from z-scores [22] for comparison of the AUC values between the three predictive models. However, there was no significant difference in the AUC values between the three predicting models (model 1 vs. model 2, z-score, 0.078, *p*-value, 0.94; model 1 vs. model 3, z-score, −0.125, *p*-value, 0.90; model 2 vs. model 3, z-score, −0.203, *p*-value, 0.84). The mean GVIF values of the three predictive models ranged from 1.022 to 1.138 where multicollinearity was not detected (Table 3). The ORs of selected variables in model 1, model 2, and model 3 are shown in the Supplementary Materials (Table S1).

## 4. Discussion

This study investigated the status of KMHC use to treat FD and analyzed the predisposing, enabling, and need factors that affect it based on Andersen’s behavior model using KHPS 2017 data. We used these data because they are nationally representative estimates of health care use, medical expenditures, and factors affecting the use of medical facilities and medical expenditures in Korea [14]. Studies have used these data to measure the association between unmet needs and health-related quality of life [23] and outpatient expenditure trends of patients diagnosed with lumbar intervertebral disc herniation [24].

Our results show that after adjusting for all predisposing, enabling, and need factors, there was a significantly higher probability of KMHC use to treat FD in patients who were younger, residing in Busan, Daegu, Ulsan, or Gyeongsang, and had private health insurance. In addition, sex, age, private health insurance, and stress were the most predictive variables for KMHC use in patients with FD. This means that younger women who are privately insured and frequently or always under stress are more likely to use KMHC to treat FD.

In particular, it was found that female patients with FD used KMHC more than men. This is similar to a study that analyzed the herbal medicine prescription status of patients with FD through Korean national health insurance claims data from the Health Insurance Review and Assessment Service [11]. The results of our study are predictable because the worldwide prevalence of FD has been reported to be higher in women [3,25], and trust and use of herbal medicines are also higher among women [26].

Meanwhile, our study found that elderly patients with FD had relatively little use of KMHC. Considering the high preference for complementary and integrative medicine among the elderly, these results of our study are unexpected [26,27]. Conflicting results have been reported in previous studies assessing KMHC use and patient age. While most studies report that older people use KM more [11], some studies have reported that the demand for KM is high in younger age groups [28]. Moreover, some studies have reported that it is independent of age [29]. However, there are differences in the data source, disease, and number of samples between individual studies. Additionally, the number of samples included in this study was relatively small. Therefore, the relationship between KMHC use and sex in patients with FD needs to be confirmed through further studies.

Patients living in Busan, Daegu, Ulsan, or Gyeongsang had a relatively higher rate of KMHC use compared with patients living in Seoul, Gyeonggi, or Incheon. Interestingly, the total number of KMHC users was highest in Seoul, Gyeonggi, and Incheon. This is similar to the results of a previous study that showed a high proportion of patients using KMHC were living in Gyeongsang-do when compared with other regions; however, there was no significant difference in the number of patients using KMHC between regions [30]. Our findings may be due to the differences in the geographical locations of these areas. In particular, herbal medicine materials often grow and are easily found in mountainous areas, and the geographical location of these cities (Busan, Daegu, Ulsan, or Gyeongsang) has more mountains around them than the locations of other cities. In Daegu in particular, there is a wholesale herbal medicine market with high-quality herbal medicines and clinics. For these reasons, access to KMHC in these cities may have been increased. However, to the best of our knowledge, no previous epidemiological studies have measured the differences in the regional distribution of patients with FD and medical service utilization preferences. Therefore, future social scientific research should be conducted to assess this further.

We found that KMHC use in patients with private health insurance was significantly higher. Moreover, private health insurance was a significant factor affecting KMHC use to treat FD. This result was obtained after adjusting for enabling factors such as household income level, employment status, health insurance type, and number of household members. Although several hypotheses, such as adverse selection and moral hazard, have been proposed for the increase in health care use behavior of insured persons due to private health insurance [31], these hypotheses could not be confirmed in this study, and additional research should be conducted in this regard. In the past, private health insurance consisted of fixed benefit insurance; however, indemnity insurance is more prevalent in recent years. The expansion of the private health insurance market has led to a trend toward indemnity coverage for various diseases, including FD, rather than fixed coverage for serious diseases. In particular, indemnity insurance is associated with an increase in health care use [32,33], which is consistent with the results of our study.

The health insurance market in the Republic of Korea is composed of national health insurance, which is subscribed to by all citizens, and optional private health insurance. The Korean medical system is divided into conventional Western medicine and KM. However, KM accounts for a small percentage of the total medical service use. Therefore, the proportion of KM covered by health insurance benefits is relatively low. This indicates that the role of private health insurance is considered relatively important in KM institutions [30]. In addition, a survey of the general public on the use of KM has reported that the expansion of insurance benefit coverage was the highest priority for improvement in the KM field in the future [34]. This was particularly important for the treatment of FD in KM clinical settings because Liujunzi-tang (Yukgunja-tang), Zhishixiaopi-wan (Jisilsobi-hwan), and other herbal decoctions that are not covered by the NHIS in Korea are actively used [35]. The results of our study suggest that the KMHC use for FD in Korea is strongly influenced by private health insurance, which is dependent on an individual’s economic power. This means that even though many people use KMHC to treat and manage FD [8], it is not sufficiently covered by public health insurance. Korean health policies should be adjusted accordingly to expand the national health insurance coverage for KM treatment to alleviate the imbalance in medical service accessibility for FD. This may initially increase the financial burden on the KMHC system. However, such a policy will improve access for patients with FD to receive appropriate treatment at an early stage, thereby minimizing direct and indirect economic impacts such as loss of productivity due to FD symptoms, and ultimately reducing the socioeconomic burden.

High stress levels were found to affect KMHC use in patients with FD. Higher levels of perceived stress increase the risk of dyspepsia [36,37]. Additionally, stress is associated with more clinic visits and health care use [38,39]. KM treatment improves FD symptoms and psychological variables, such as anxiety, depressed mood, stress, and emotional state [40]. Our findings suggest that KM clinic visits could be increased to manage both dyspepsia symptoms and stress in patients with high levels of stress. This indicates that KM clinicians and caregivers should consider the importance of identifying and managing patient stress levels when treating FD.

The limitations of our study are as follows. First, although trained investigators collected data using CAPI, a recall bias might exist in that some self-reported data were analyzed, rather than data from medical records. Second, the number of patients using KMHC included in the analysis was relatively small. In addition, although KHPS is a longitudinal data source, a longitudinal analysis could not be performed in this study because of the small number of consecutive episodes of health care use for FD treatment. Therefore, it will be necessary to reconfirm the factors affecting KMHC use derived in this study in a larger sample of patients with FD. In addition, it would be helpful to conduct longitudinal studies to investigate the predictors of KMHC use in patients with FD to gain insights into the causal relationship of health care use.

This is the first study to identify the factors affecting the use of KMHC in patients with FD. This study is important for clinicians, researchers, and policymakers who need to ascertain the status of KMHC use in patients with FD and identify approaches related to their health care behavior. Through this, it will contribute to the development and supplementation of a system that can help promote individual health and improve the financial efficiency of the health care system.

## 5. Conclusions

Participants aged 65 years or older were less likely to use KMHC to treat FD than those aged 19 to 34 years.

Participants with private health insurance were more likely to use KMHC to treat FD than those without private health insurance.

Frequently or always stressed participants were more likely to use KMHC to treat FD, although this relationship was not statistically significant.

## Figures and Tables

**Table 1:** General participant characteristics.

Variables			KMHC Use of Functional Dyspepsia				*p*-Value
			Non-Use		Use		
	Total	%	N	%	N	%	
Number of participants	404		359		45		
**Predisposing factors**							
Sex							0.16
Men	132	32.67	122	33.98	10	22.22	
Women	272	67.33	237	66.02	35	77.78	
Age (years)							<0.001
19–34	30	7.43	22	6.13	8	17.78	
35–49	72	17.82	61	16.99	11	24.44	
50–64	102	25.25	86	23.96	16	35.56	
65 or older	200	49.50	190	52.92	10	22.22	
Education							<0.01
Elementary school or below	139	34.41	132	36.77	7	15.56	
Middle/high school	190	47.03	166	46.24	24	53.33	
College or above	75	18.56	61	16.99	14	31.11	
Region							0.15 †
Seoul/Gyeonggi/Incheon	163	40.35	145	40.39	18	40.00	
Gangwon	35	8.66	32	8.91	3	6.67	
Daejeon/Chungcheong/Sejong	69	17.08	63	17.55	6	13.33	
Gwangju/Jeolla/Jeju	48	11.88	46	12.81	2	4.44	
Busan/Daegu/Ulsan/Gyeongsang	89	22.03	73	20.33	16	35.56	
**Enabling factors**							
Household income							<0.01 †
1st quintile (lowest)	97	24.01	94	26.18	3	6.67	
2nd quintile	99	24.50	93	25.91	6	13.33	
3rd quintile	70	17.33	60	16.71	10	22.22	
4th quintile	81	20.05	67	18.66	14	31.11	
5th quintile (highest)	57	14.11	45	12.53	12	26.67	
Employment status							0.19 †
Unemployed	226	55.94	206	57.38	20	44.44	
Employed	138	34.16	117	32.59	21	46.67	
Self-employed	40	9.90	36	10.03	4	8.89	
Health insurance type							0.24 †
Employee health insurance	296	73.27	259	72.14	37	82.22	
Local subscriber	73	18.07	66	18.38	7	15.56	
Medical aid or others	35	8.66	34	9.47	1	2.22	
Private health insurance							<0.01
No	137	33.91	131	36.49	6	13.33	
Yes	267	66.09	228	63.51	39	86.67	
Number of household members							0.34
1	64	16.70	58	16.16	6	13.33	
2	163	42.72	149	41.50	14	31.11	
3	67	17.23	59	16.43	8	17.78	
4 or more	110	23.34	93	25.91	17	37.78	
**Need factor**							
Disability							0.40 †
No	370	91.58	327	91.09	43	95.56	
Yes	34	8.42	32	8.91	2	4.44	
Self-assessed health							0.08
Poor	95	23.51	87	24.23	8	17.78	
Fair	180	44.55	164	45.68	16	35.56	
Good	129	31.93	108	30.08	21	46.67	
Number of chronic diseases							<0.05 †
0	140	34.65	116	32.31	24	53.33	
1	90	22.28	82	22.84	8	17.78	
2	84	20.79	76	21.17	8	17.78	
3 or more	90	22.28	85	23.68	5	11.11	
Depressed mood							0.76 †
No	374	92.57	333	92.76	41	91.11	
Yes	30	7.43	26	7.24	4	8.89	
Stress							0.10 †
Never or rarely	324	80.20	287	79.94	37	82.22	
Sometimes	49	12.13	47	13.09	2	4.44	
Frequently or always	31	7.67	25	6.96	6	13.33	
BMI (kg/m^2^)							0.61 †
<18.5	21	5.20	19	5.29	2	4.44	
18.5–22.9	179	44.31	154	42.90	25	55.56	
23.0–24.9	117	28.96	107	29.81	10	22.22	
25.0–29.9	78	19.31	71	19.78	7	15.56	
≥30	9	2.23	8	2.23	1	2.22	
Smoking							0.24 †
Never smoked	300	74.26	262	72.98	38	84.44	
Quit smoking	71	17.57	67	18.66	4	8.89	
Smoking	33	8.17	30	8.36	3	6.67	
Drinking							0.29 †
Never drunk	136	33.66	119	33.15	17	37.78	
Monthly or less	146	36.14	132	36.77	14	31.11	
2 to 4 times a month	69	17.08	58	16.16	11	24.44	
2 times a week or more	53	13.12	50	13.93	3	6.67	
Physical activities							0.52
Not at all	273	67.57	245	68.25	28	62.22	
Once a week or more	131	32.43	114	31.75	17	37.78	

Abbreviations: BMI, body mass index; KMHC, Korean medicine health care; N, number of participants. *p*-values were obtained from chi-squared tests, and *p*-values † were obtained from Fisher’s exact tests.

**Table 2:** Factors affecting KMHC use for functional dyspepsia.

Variables	Crude		Adjusted 1		Adjusted 2		Adjusted 3	
	OR (95% CI)	*p*-Value	OR (95% CI)	*p*-Value	OR (95% CI)	*p*-Value	OR (95% CI)	*p*-Value
**Predisposing factors**								
Sex								
Men	1		1		1		1	
Women	1.8 (0.86–3.76)	0.12	1.78 (0.82–3.88)	0.15	1.86 (0.79–4.39)	0.16	1.82 (0.47–7.01)	0.38
Age (years)								
19–34	1		1		1		1	
35–49	0.5 (0.18–1.39)	0.18	0.44 (0.15–1.31)	0.14	0.39 (0.13–1.22)	0.11	0.42 (0.11–1.55)	0.19
50–64	0.51 (0.19–1.35)	0.16	0.52 (0.18–1.52)	0.23	0.4 (0.12–1.3)	0.13	0.26 (0.06–1.1)	0.07
65 or older	0.14 (0.05–0.4)	<0.001	0.19 (0.05–0.7)	<0.05	0.23 (0.05–1.1)	0.07	0.14 (0.02–0.93)	<0.05
Education								
Elementary or below	1		1		1		1	
Middle/high school	2.73 (1.14–6.52)	<0.05	1.7 (0.6–4.79)	0.31	1.53 (0.51–4.53)	0.45	1.98 (0.58–6.83)	0.28
College or above	4.33 (1.66–11.26)	<0.01	1.98 (0.56–7.03)	0.29	1.74 (0.46–6.55)	0.41	2.38 (0.54–10.43)	0.25
Region								
Seoul/Gyeonggi/Incheon	1		1		1		1	
Gangwon	0.76 (0.21–2.72)	0.67	0.98 (0.26–3.69)	0.97	0.85 (0.21–3.45)	0.82	0.94 (0.21–4.16)	0.93
Daejeon/Chungcheong/Sejong	0.77 (0.29–2.02)	0.59	1.22 (0.44–3.4)	0.70	1.48 (0.5–4.35)	0.48	1.36 (0.41–4.53)	0.61
Gwangju/Jeolla/Jeju	0.35 (0.08–1.57)	0.17	0.49 (0.11–2.28)	0.37	0.55 (0.11–2.66)	0.46	0.44 (0.08–2.46)	0.35
Busan/Daegu/Ulsan/Gyeongsang	1.76 (0.85–3.66)	0.13	2.29 (1.06–4.92)	<0.05	2.53 (1.14–5.61)	<0.05	2.45 (1.02–5.88)	<0.05
**Enabling factors**								
Household income								
1st quintile (lowest)	1				1		1	
2nd quintile	2.02 (0.49–8.32)	0.33			1.15(0.23–5.62)	0.87	1.11 (0.2–6.27)	0.91
3rd quintile	5.22 (1.38–19.76)	<0.05			3 (0.62–14.61)	0.17	2.72 (0.5–14.77)	0.25
4th quintile	6.55 (1.81–23.68)	<0.01			2.98 (0.56–15.99)	0.20	3.13 (0.51–19.16)	0.22
5th quintile (highest)	8.36 (2.25–31.07)	<0.01			4.19 (0.73–23.87)	0.11	4 (0.6–26.45)	0.15
Employment status								
Unemployed	1				1		1	
Employed	1.85 (0.96–3.55)	0.07			0.97 (0.43–2.19)	0.95	0.89 (0.37–2.18)	0.80
Self-employed	1.14 (0.37–3.55)	0.82			2.2 (0.6–8.11)	0.24	1.85 (0.42–8.11)	0.42
Health insurance type								
Employee health insurance	1				1		1	
Local-subscriber health insurance	0.74 (0.32–1.74)	0.49			1 (0.39–2.61)	0.99	0.95 (0.34–2.71)	0.93
Medical aid or others	0.21 (0.03–1.55)	0.13			0.44 (0.05–3.97)	0.46	0.3 (0.03–3.51)	0.34
Private health insurance								
No	1				1		1	
Yes	3.74 (1.54–9.06)	<0.01			2.09 (0.72–6.06)	0.17	3.41 (1.02–11.42)	<0.05
Number of household members								
1	1				1		1	
2	0.91 (0.33–2.48)	0.85			0.65 (0.2–2.14)	0.48	0.53 (0.14–1.91)	0.33
3	1.31 (0.43–4.02)	0.64			0.32 (0.08–1.3)	0.11	0.26 (0.05–1.22)	0.09
4 or more	1.77 (0.66–4.73)	0.26			0.35 (0.09–1.28)	0.11	0.28 (0.07–1.17)	0.08
**Need factor**								
Disability								
No	1						1	
Yes	0.48 (0.11–2.05)	0.32					1.19 (0.17–8.2)	0.86
Self-assessed health								
Poor	1						1	
Fair	1.06 (0.44–2.58)	0.90					0.72 (0.23–2.26)	0.57
Good	2.11 (0.89–5.01)	0.09					1.07 (0.32–3.53)	0.92
Number of chronic diseases								
0	1						1	
1	0.47 (0.2–1.1)	0.08					0.98 (0.34–2.88)	0.98
2	0.51 (0.22–1.19)	0.12					1.71 (0.49–6.03)	0.40
3 or more	0.28 (0.1–0.78)	<0.01					0.94 (0.22–4.03)	0.93
Depressed mood								
No	1						1	
Yes	1.25 (0.42–3.76)	0.69					2.39 (0.56–10.16)	0.24
Stress								
Never or rarely	1						1	
Sometimes	0.33 (0.08–1.42)	0.14					0.26 (0.05–1.37)	0.11
Frequently or always	1.86 (0.72–4.83)	0.20					3.26 (0.85–12.51)	0.09
BMI (kg/m2)								
<18.5	1						1	
18.5–22.9	1.54 (0.34–7.03)	0.58					1.35 (0.22–8.18)	0.74
23.0–24.9	0.89 (0.18–4.38)	0.88					0.87 (0.13–5.97)	0.88
25.0–29.9	0.94 (0.18–4.88)	0.94					1.07 (0.15–7.52)	0.95
≥30	1.19 (0.09–15.03)	0.89					0.96 (0.03–27)	0.98
Smoking								
Never smoked	1						1	
Quit smoking	0.41 (0.14–1.19)	0.10					1.46 (0.24–8.96)	0.68
Smoking	0.69 (0.2–2.37)	0.56					1 (0.14–7.13)	0.99
Drinking								
Never drunk	1						1	
Monthly or less	0.74 (0.35–1.57)	0.44					0.56 (0.22–1.46)	0.24
2 to 4 times a month	1.33 (0.58–3.02)	0.50					0.63 (0.21–1.9)	0.41
2 times a week or more	0.42 (0.12–1.49)	0.18					0.28 (0.05–1.47)	0.13
Physical activities								
Not at all	1						1	
Once a week or more	1.3 (0.69–2.48)	0.42					0.69 (0.31–1.56)	0.38
Mean GVIF			1.161		1.291		1.547	

Abbreviations: BMI, body mass index; CI, confidence interval; mean GVIF, mean of the generalized variance inflation factors; KMHC, Korean medicine health care; OR, odds ratio. *p*-values in crude were obtained from simple logistic regressions for each variable. *p*-values in adjusted 1 were obtained from multiple logistic regression for all the predisposing factors. *p*-values in adjusted 2 were obtained from multiple logistic regression for all the predisposing and enabling factors. *p*-values in adjusted 3 were obtained from multiple logistic regression for all the predisposing, enabling, and need factors.

**Table 3:** Predictive powers of the predisposing, enabling, and need factors for KMHC use to treat functional dyspepsia.

Model	Factor	Selected Variables	Mean GVIF	AUC (95% CI)	AIC
Model 1	Predisposing	Sex, Age, Region	1.022	0.701 (0.626–0.777)	273.127
Model 2	Predisposing + Enabling	Sex, Age, Region, Private health insurance	1.088	0.696 (0.623–0.768)	272.558
Model 3	Predisposing + Enabling + Need	Sex, Age, Private health insurance, Stress	1.138	0.709 (0.637–0.781)	264.762

Abbreviations: AIC, Akaike information criterion; AUC, areas under the receiver operating characteristic curve; CI, confidence interval; mean GVIF, mean of generalized variance inflation factors; KMHC, Korean medicine health care. The variables in model 1 were selected by applying a stepwise procedure to multiple logistic regression model for predisposing factors. The variables in model 2 were selected by applying a stepwise procedure to multiple logistic regression model for predisposing and enabling factors. The variables in model 3 were selected by applying a stepwise procedure to multiple logistic regression model for predisposing, enabling, and need factors.

## Data Availability

The datasets used or analyzed during the current study are available from the corresponding author upon reasonable request.

## Supplementary Materials

The following supporting information can be downloaded at: https://www.mdpi.com/article/10.3390/healthcare10071192/s1, Table S1: Odds ratios of selected variables.
