# Supplementary material for: Factors Affecting Korean Medicine Health Care Use for Functional Dyspepsia: Analysis of the Korea Health Panel Survey 2017
Source: Healthcare (Basel). 2022 Jun 25;10(7):1192. doi: 10.3390/healthcare10071192 (PMC9320755; doi:10.3390/healthcare10071192)
Supplement: Supplementary file 1 [file healthcare-10-01192-s001.zip › healthcare-1771367-SI.pdf]

Table S1. Odds ratios of selected variables.

Selected Variables	Model 1		Model 2		Model 3	
	OR (95% CI)	*p*-value	OR (95% CI)	*p*-value	OR (95% CI)	*p*-value
Predisposing factors						
Sex						
Men	1		1		1	
Women	1.67 (0.78-3.59)	0.19	1.64 (0.76-3.55)	0.21	2 (0.91-4.36)	0.08
Age (years)						
19-34	1		1		1	
35-49	0.43 (0.15-1.23)	0.11	0.37 (0.13-1.1)	0.07	0.37 (0.12-1.08)	0.07
50-64	0.45 (0.17-1.23)	0.12	0.42 (0.15-1.14)	0.09	0.31 (0.11-0.88)	<0.05
Over 65	0.13 (0.04-0.37)	<0.001	0.16 (0.05-0.48)	<0.01	0.14 (0.05-0.42)	<0.01
Region						
Seoul/Gyeonggi/Incheon	1		1			
Gangwon	0.87 (0.23-3.22)	0.83	0.86 (0.23-3.22)	0.83		
Daejeon/Chungcheong/Sejong	1.23 (0.44-3.41)	0.70	1.32 (0.47-3.71)	0.59		
Gwangju/Jeolla/Jeju	0.48 (0.1-2.21)	0.35	0.46 (0.1-2.14)	0.32		
Busan/Daegu/Ulsan/Gyeongsang	2.27 (1.06-4.87)	<0.05	2.21 (1.03-4.75)	<0.05		
Enabling factors						
Private health insurance						
No			1		1	
Yes			2.19 (0.81-5.97)	0.12	2.96 (1.04-8.4)	<0.05
Need factor						
Stress						
Never or rarely					1	
Sometimes					0.24 (0.05-1.1)	0.07
Frequently or always					4.04 (1.35-12.12)	<0.05
Mean VIF	1.022		1.088		1.138	

Abbreviations: CI, confidence interval; Mean GVIF, the mean of generalized variance inflation factors; KMHC, Korean medicine health care; OR, odds ratio. The variables and *p*-values in Model 1 were obtained by applying stepwise procedures to multiple logistic models for predisposition factors. The variables and *p*-values in Model 2 were obtained by applying stepwise procedures to multiple logistic models for predisposition and enabling factors. The variables and *p*-values in Model 3 were obtained by applying stepwise procedures to multiple logistic models for predisposition, enabling, and need factors.
